# Antimicrobial Meshes for Hernia Repair: Current Progress and Perspectives

**DOI:** 10.3390/jcm11030883

**Published:** 2022-02-08

**Authors:** Simona Mirel, Alexandra Pusta, Mihaela Moldovan, Septimiu Moldovan

**Affiliations:** 1 Department of Medical Devices, Iuliu Hațieganu University of Medicine and Pharmacy, 400349 Cluj-Napoca, Romania; smirel@umfcluj.ro; 2 Pediatric Surgery Department, Emergency Clinical Children’s Hospital, 400370 Cluj-Napoca, Romania; mihaelamirel@gmail.com; 3 Surgery Department, Prof. Dr. O. Fodor Regional Institute of Gastroenterology and Hepatology, 400162 Cluj-Napoca, Romania; septimiu1995@yahoo.com

**Keywords:** hernia, infection, biomaterials, antimicrobial surgical meshes, antimicrobial metals, antibiotics, antiseptics

## Abstract

Recent advances in the development of biomaterials have given rise to new options for surgery. New-generation medical devices can control chemical breakdown and resorption, prevent post-operative adhesion, and stimulate tissue regeneration. For the fabrication of medical devices, numerous biomaterials can be employed, including non-degradable biomaterials (silicone, polypropylene, expanded polytetrafluoroethylene) or biodegradable polymers, including implants and three-dimensional scaffolds for tissue engineering, which require particular physicochemical and biological properties. Based on the combination of new generation technologies and cell-based therapies, the biocompatible and bioactive properties of some of these medical products can lead to progress in the repair of injured or harmed tissue and in tissue regeneration. An important aspect in the use of these prosthetic devices is the associated infection risk, due to the medical complications and socio-economic impact. This paper provides the latest achievements in the field of antimicrobial surgical meshes for hernia repair and discusses the perspectives in the development of these innovative biomaterials.

## 1. Introduction

Based on the combination of new generation technologies and cell-based therapies, the biocompatible and bioactive properties of medical products can lead to progress in repairing injured or harmed tissues and in tissue regeneration. One of the most common abdominal wall defects with an indication for surgery is represented by hernias, a protrusion of an organ outside its cavity through an area of low resistance [1]. It is considered that the main cause of hernia is related to collagen disorders as well as to the tension surgical technique used. Damage of the tissues leads to fibrosis, which does not have the same mechanical strength to keep the integrity of the wall when subject to sustained pressure exerted from inside [2]. Nowadays, mesh prostheses and no-tension surgical techniques have become a gold standard in treating hernias. Taking into account the high recurrence rates, surgical meshes are essential medical devices that support the damaged tissue and its healing. Surgical prostheses for hernia repair aim to strengthen and replace tissue defects, stabilizing the abdominal wall and providing long-term resistance [3]. The importance of meshes in hernia repair treatment is highlighted by the increasing number of patent publications in the field of hernia repair (56 patents reported from 2008 to 2018) [4]. Different surgical meshes for hernia exist with respect to their composition and properties. The composition of the material and its structure can significantly affect the biocompatibility of the prosthetic device [5], but the mechanical resistance of wall reconstruction is reported to be similar, independently of the material used. In order to optimize the surgical handling of the prosthetic devices, their good flexibility and memory are important, because they provide better adaptability to the mesh [6]. As a typical biomaterial-based implantable device, the optimal mesh should not only have the general qualities of biomaterials (good tissue tolerance, non-migration, chemical, and mechanical stability), but also claim other performance characteristics: resist shrinkage, block transmission of infectious diseases, good tissue integration, and minimal adhesion formation.

The biomaterials used for hernia meshes include a wide range of natural and synthetic polymers, with different structures (reticular, laminar, hybrid) and characteristics (pore size, filament distribution). First-generation surgical meshes—based mainly on polypropylene (PP), have as their main advantage the high tensile strength (necessary to support intra-abdominal pressure), but also disadvantages related to recurrence, adhesion, and infections at the hernia site. The second generation of these medical devices proposes composite systems, combining various materials, and offers minimal adhesion formation. Biological materials represent the third generation of these prosthetic devices, being designed as a matrix for native cells to generate connective tissue that should replace the tissue in the defect of the wall. The main advantage of these devices is their superior biocompatibility, respectively the lack of inflammatory response from the body, while the main disadvantage is their higher cost compared to other types available [7,8].

Nowadays, there is a broad spectrum of synthetic prosthetic biomaterials (absorbable, non-absorbable) or biological meshes (xenografts and allografts) that hernia treatment may benefit from, which have been the subject of some reviews in recent years [1,3,9,10,11,12,13,14,15].

An important risk associated with the use of these prostheses is represented by the associated infections, due to the post-operative complications that affect the patients’ quality of life and have a high socio-economic impact [16,17]. In this context, the development of surgical meshes with antimicrobial properties is vital for the prevention of surgical-site infections.

**Figure 1:**
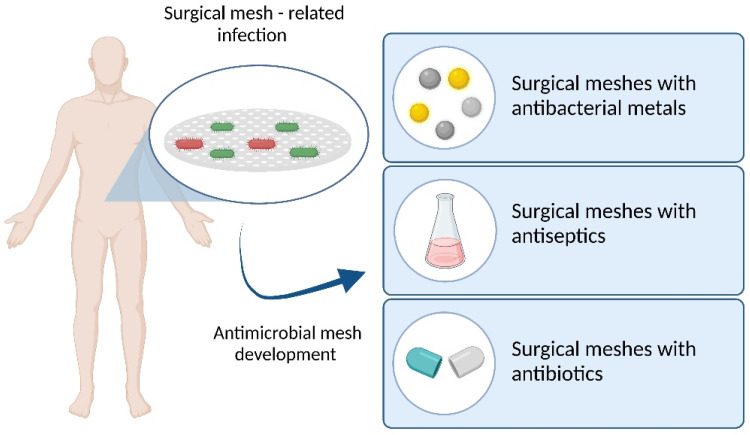
Strategies for the development of antimicrobial meshes. Created with BioRender.com.

In this review, the latest achievements in the field of antimicrobial surgical meshes are presented and discussed. The burden of mesh-related infections is highlighted and different types of meshes with antimicrobial agents are presented, together with their advantages and disadvantages. The meshes presented in this work are divided into three categories: meshes with antimicrobial metals, meshes with antiseptics, and meshes with antibiotics (Figure 1).

## 2. Prosthetic Materials for Clinical Use

During the past few decades, the use of non-absorbable prostheses has been well established in the surgical treatment of hernias. Non-absorbable biomaterials commonly used include polypropylene PP, poly(ethylene terephthalate) (PET) and expanded poly-tetrafluoroethylene (ePTFE) [18]. Polypropylene meshes are considered the gold standard of prosthetic materials used in hernia surgery [11] with the advantage of parietal host tissue in-growth, but with the disadvantage of forming adhesions with tissues and organs. In contrast, ePTFE meshes are better at preventing adhesion formation but are less capable of promoting host tissue in-growth [19]. PET meshes present better histocompatibility with less foreign body response, with strong promotion of host tissue in-growth, but with long-term stability and possible infection risk remaining important concerns [20,21].

Absorbable meshes were created in order to reduce the foreign body response, more commonly seen in permanent prostheses [9]. Absorbable meshes seem a better option, as they reduce some of the disadvantages of non-absorbable ones, such as the risk of infection in pre-contaminated areas, decreased normal tissue growth, fistula formation, chronic pain, and movement restriction [22,23]. For example, the multifilament meshes made of poly (glycolic) acid (PGA), have minimal foreign body response and minimal adhesion formation. On the other hand, they present a rapid degradation index resulting in insufficient and unstable collagen formation with a high recurrence rate of hernia [9,21,24]. The multifilament mesh made of polyglactin 910 (92% glycolide, 8% lactide) has an improved short-term mechanical stability, but without sufficient stability in the longer term and it also has a higher inflammatory response and fibrosis formation rate [9,23,25]. Polylactide mesh (95% lactide, 5% glycolide) is a multifilament mesh with further improved stability, reduced seroma formation, and decreased risk of infection, with the drawback of increased foreign body response [23]. An innovative macroporous mesh with combined fast degrading and slow degrading [(poly(lactide-co-glycolide acid)-poly(trimethylene carbonate)] fibers (PLGA-PTMC) was obtained by knitting two resorbable filaments, a slow and a fast degrading one, into a multifilament mesh. This comes with the advantage of preserving mechanical stability with more physiological collagen formation and better biointegration [26,27]. A PGA-PTMC (polyglycolic acid-poly(trimethylene carbonate) copolymer poses the same concern of long-term mechanical strength as the other absorbable prostheses but comes with optimized tissue in-growth and better resistance to infection [9,28,29]. A monofilament poly(4-hydroxybutyrate) mesh, made from a natural polymer, provides short-term mechanical strength, resistance to infections, but also its disadvantages are long degradation time (more than 72 weeks) and limited information regarding long-term host response [30].

Biologically derived meshes were developed for the purpose of covering abdominal wall defects when there is contamination or highly probable contamination [18]. In essence, biological meshes are extracellular matrices that pass through a process of decellularization in order to act as active scaffolds [31]. The process of biointegration includes the promotion of collagen formation, tissue in-growth, and neovascularization, resulting in a newly formed tissue that can sustain and offer integrity to the abdominal wall. Biological prostheses with medical use are classified as xenogenic (of animal origin) or allogenic (from human cadavers) [9,21].

As xenogenic prostheses, the most commonly used are small intestine submucosa meshes and acellular porcine derma tissue meshes. Thus, falling into the first group, a commercially available mesh consists of four layers of mostly acellular extracellular matrix. It enhances neovascularization and tissue formation, being also useful in contaminated areas by decreasing the risk of infection [25,32]. Another currently used biological mesh is formed by crosslinking small intestine submucosa with 1-ethyl-3(3-dimethylaminopropyl)-carbodiimide hydrochloride (EDC) and could be useful in surgery. A disadvantage would be inducing foreign body response due to crosslinking [33]. Falling into the second group (acellular porcine derma tissue meshes), another prosthetic device has been developed by crosslinking acellular porcine derma with hexamethylene diisocyanate (HMDI), which adds stability by inhibition of collagen degradation. It presents a low foreign body response, with little adhesion formation, also promoting neoangiogenesis and tissue in-growth [9,34,35]. Another clinically used mesh uses crosslinking with 1-ethyl-3-carbodiimide hydrochloride (EDAC) for the same purposes. Studies conducted showed higher stability and lower infection rates compared to the HMDI-crosslinked mesh. However, both of them demonstrated low tissue in-growth index [36]. A commercially available mesh made from the acellular porcine dermis without crosslinking offered better tissue integration but has shown mechanical instability compared to EDAC and HMDI- crosslinked meshes [37].

## 3. Surgical Mesh Related Infection

Mesh-related infections could occur weeks or years after surgical intervention and differ from incisional surgical site infections, occurring superficially, within 30 days of the intervention [38]. The data reported for the incidence differs from 1 to 8%, influenced not only by the mesh type but also by patients’ co-morbidities (including diabetes and obesity), surgical technique, or strategy for prevention [38,39,40]. The most common microorganisms related to mesh infection are *Staphylococcus aureus* and *Staphylococcus epidermidis*, but infections with *Pseudomonas aeruginosa*, *Streptococcus pyogenes*, *Escherichia coli*, *Klebsiella pneumoniae*, *Enterococcus faecalis*, or *Candida albicans* have also been reported.

Prosthetic devices are generally made out of materials that do not possess intrinsic antimicrobial activity, thus justifying the infection risk associated with the use of these medical devices. The results concerning the influence of mesh type on the incidence of mesh-related infection showed that absorbable synthetic materials are generally more susceptible to bacterial colonization than non-absorbable materials [10,41]. The reported data also showed that the risk of infection is mainly determined by the type of filament. Generally, the use of multifilament polyester mesh has a higher incidence of infection than other types of meshes, while the meshes with the lowest risk are made with monofilament [39].

A comparative in vitro study between eight different types of meshes available for clinical use, based on PP, PET, polyglactin 910 (PG-910), ePTFE, and condensed polytetrafluoroethylene (cPTFE) demonstrated the influence of polymer type, but also of certain morphological characteristics of synthetic materials tested. After being inoculated with *Staphylococcus aureus* and *Staphylococcus epidermidis*, the results showed that ePTFE had significantly higher rates of bacterial adherence compared to the other tested polymers. The results highlighted that multifilament meshes had significantly greater bacterial adherence with both pathogens compared to all of the monofilament meshes. The larger filament diameter and smaller pores increased the bacterial adherence. The comparison between coated and uncoated meshes showed that there was no significant difference between titanium-coated PP and uncoated PP, while silver-chlorhexidine coating significantly reduced the bacterial adherence, highlighting the role of the antiseptic agent [42].

## 4. Types of Antimicrobial Surgical Meshes

The strategies for obtaining antimicrobial meshes involve the physical coating or chemical functionalization by introducing different antiseptic/antibacterial agents [43]. The prosthetic devices with prophylactic antimicrobial activity have the surface modified with anti-adhesive substances, metal coatings, or various antimicrobial agents.

The commercially available products in clinical use are coated meshes (with silver, chlorhexidine, or their different mixtures), that demonstrated a significant antibacterial activity with reduced bacterial adhesion [42,44], but more antimicrobial meshes coated with antimicrobial compounds are still under research.

### 4.1. Surgical Meshes with Antimicrobial Metals

The antibacterial role of silver is recognized and this metal has been widely used to confer antimicrobial properties to various devices [45,46,47]. Their anti-infective activity is conferred by the Ag^+^ cations released from the prosthetic material. As it is known, silver is biologically active only in its soluble form, and not as a chemical complex. For ordinary silver formulations such as silver nitrate or silver sulfadiazine, the released silver ions are rapidly inactivated by chemical reactions with chloride or organic ions. The use of nanocrystalline silver particles (having better antibacterial properties compared to those of ordinary silver due to their size and surface area) offers a sustained antimicrobial activity [48]. The use of metal coating as an alternative to antibiotic-coated prosthetic devices has been proposed to decrease the incidence of prosthetic mesh infections, which could lead to significant enhancements in antimicrobial activity.

Nowadays, the commercially available products clinically used are PP meshes coated with Ag^+^, but more antimicrobial meshes coated with antimicrobial metals (Ag, Zn, Pd, Au) are still under research (Table 1).

Polypropylene meshes with a silver layer could be a viable replacement for normal PP meshes, providing better prevention of infections. Generally, the meshes were coated by magnetron sputtering technique or by using physical vapor deposition. The effectiveness against bacterial infection was evaluated comparing uncoated vs. silver-coated implants in vitro and in vivo studies. Thus, in vitro results demonstrate a significant bactericidal efficacy against *S. aureus* of nanocrystalline silver-coated surgical mesh, highlighting a direct proportionality ratio between the diameter of the inhibition zone (ZOI) and silver concentration from meshes [49]. Badiou et al. demonstrated for the first time the in vitro and in vivo antibacterial efficacy of silver-coated PP mesh to prevent infection in animal hernia repair [51].

An in vivo study regarding the efficacy of nanocrystalline silver-coated PP meshes against methicillin-resistant *Staphylococcus aureus* (MRSA)-induced infection in a rat model showed a significantly better bactericidal effect than the non-coated PP device [50].

Considering the antimicrobial activity and the anti-biofilm properties of the biomaterials used, the strategy to reduce nosocomial infections related to prosthetic meshes includes the prevention of microbial colonization. To this aim, recent studies focused on preventing biofilm formation. Thus, a new prosthetic mesh—a PP mesh coated with metal-containing diamond-like carbon (Me-DLC) thin films—was obtained. The results showed that only silver and cobalt in Me-DLC coated meshes have exhibited inhibition of growth of all tested bacterial strains [55].

A mesh composed of two PP layers (macroporous light mesh and thin transparent film) coated with a thin layer of Ag/SiO_2_-nanoclusters was characterized and highlighted cell growth and antibacterial properties [52].

Composite materials were also used for this purpose. A nano-silver composite mesh was proposed by Zhang et al. [53]. Their composite materials were based on polyethyleneglycol (PEG) and hydrogel to reduce adhesion, while flexible polyurethane (PU) nanofibers were used to offer superior mechanical properties. In addition, due to the presence of silver nanoparticles, the composite mesh showed good antibacterial properties. The new mesh could be a promising option in hernia repair, due to its good integration into the adjacent abdominal wall tissue and reduced postoperative adhesion demonstrated in in vivo studies.

Nano-silver was also proposed for improving the performance of acellular and xenogenic biological materials. For example, a new mesh obtained by naturally derived biomaterial (porcine-derived small intestinal submucosa) immersed in nano-silver solution, demonstrated an excellent antibacterial effect and biosafety profile [48].

Other antimicrobial meshes coated with other antimicrobial metals (Ti, Zn, Pd, Au) were proposed. There are titanized polymeric meshes available on the market (PP filaments coated with titanium dioxide or with a layer of atomic titanium), but their antimicrobial efficacy is debatable. Thus, an in vitro study reported no significant differences in terms of bacterial adhesion between titanium-coated PP and uncoated PP. The same study showed the reduced antimicrobial activity of titanium-coated PP meshes against *S. aureus* and *S. epidermidis* compared to silver-chlorhexidine-coated meshes [42].

The antimicrobial effect against *Staphylococcus aureus* in the case of titanium coating was lower compared to that of uncoated meshes and the titanium-based mesh had no effect against *Pseudomonas aeruginosa* and *Escherichia coli* [56].

However, the antibacterial activity of titanium-modified PP meshes can be improved by vanadium doping. Vanadium-doped titanium TiO_2_ nanofilms were deposited onto PP meshes and showed high antibacterial activity against *S. aureus* and *E. coli.* The study demonstrated that V-doping leads to the formation of redox-active species which are responsible for the antibacterial effect of the meshes [58].

Zinc-impregnated meshes obtained from commercial PP mesh chemically treated with zinc (Zn^2+^) have been studied for their antimicrobial capacity. The proposed zinc-impregnated PP mesh showed better antibacterial properties compared to a commercial PP mesh when it was placed in a contaminated environment (after a follow-up of 90 days) but have led to more adhesion to viscera development than normal PP meshes [57].

Other researchers investigated the antimicrobial behavior of PP mesh coated with gold or gold-palladium (60% gold, 40% palladium). In their in vitro study on the antibacterial activity against *S. epidermidis*, the lowest bacterial growth was observed for the gold-palladium-coated devices, respectively, for gold-coated, while the highest growth of bacteria was found for the uncovered ones. The difference was obvious after 12 h, but the most significant reduction was observed after 24 h. The in vivo study also revealed significant differences between the antibacterial properties of gold-palladium-coated and gold-coated devices compared to standard PP meshes—during 24 h—the period in which the highest adhesion rate is expected [54].

A comparison between eight different commercial hernia meshes with different characteristics, based on commonly used polymers (PP, ePTFE, and cPTFE) has shown that ePTFE mesh coated with antibacterial silver chlorhexidine significantly reduced the bacterial adhesion of both *Staphylococcus aureus* and *Staphylococcus epidermidis* [42].

### 4.2. Surgical Meshes with Antiseptics

The use of antiseptics is well-documented and these substances are applied for conferring antibacterial properties to numerous medical devices (venous catheters, urinary catheters, wounds dressings). Some of the prosthetic devices available for clinical use or under research are coated/impregnated with a combination of different antimicrobials (Table 2).

Chlorhexidine (CHX) was proposed as an antiseptic agent for a new polymeric biocomposite mesh consisting of a commercially available PP mesh, impregnated with “coladerm” (biodegradable 7% poly(ester)amide ethanolic solution) and chlorhexidine. The antimicrobial efficacy of the mesh was tested in a study comprised of two parts. The in vivo experimental study comparatively evaluated the three types of meshes: PP mesh (standard), PP mesh + “coladerm”, respectively, PP mesh + “coladerm” + CHX. The clinical research results showed the antimicrobial characteristics of the new antiseptic biocomposite devices that did not lead to postoperative suppurative complications. Following the use of the new mesh, bacterial contamination was minimized to 1.26% [59].

The use of the silver/CHX combination is considered effective due to its bactericidal effect and due to preventing the proliferation of bacteria on the mesh surface. Thus, the in vitro comparison of bacterial adhesion in the case of nine meshes used in medical practice—with different structures, coated or non-coated (based on PP, PE/PEG and collagen, PP/ePTFE, PP/oxidized regenerated cellulose, PP/polyglecaprone, PP/polyglactin 910, PP/titanium coating and ePTFE with silver/chlorhexidine), showed that only ePTFE with silver chlorhexidine coating demonstrated a bactericidal effect against MRSA, due to the silver—chlorhexidine antimicrobial coating [44].

The effectiveness of silver/CHX-impregnated devices was also confirmed by an in vivo comparative study based on the affinity of *S. aureus* to different types of biomaterials (ePTFE with silver/chlorhexidine, porcine small intestinal submucosa, PP, PP/ePTFE, PP-hyaluronate/carboxymethylcellulose, or human dermal matrix) [61].

A retrospective study of patients undergoing laparoscopic ventral hernia repair using commercially available meshes confirmed that the use of antimicrobial-impregnated ePTFE mesh with silver/chlorhexidine is associated with noninfectious postoperative fever [62].

Pérez-Köhler’s group has proposed the use of CHX as an antiseptic agent, alone or in combination in several studies. In this study, a quaternary ammonium-based polymer was loaded with CHX and the polymer was subsequently coated onto PP meshes. The results indicate significant antibacterial capacity against both Gram-positive and Gram-negative bacterial strains [63].

Another experimental study evaluated the use of a CHX-loaded carboxymethylcellulose (CMC) gel in a model of *S. aureus* mesh infection. The in vitro studies confirmed the antibacterial activity and the in vivo study demonstrated that CHX incorporated in the CMC gel maintained this effect at the surgery site. A disadvantage of this approach is represented by the increased fibroblastic destruction [60].

The same group of researchers also proposed the use of a combination of CHX and allicin (a natural antibacterial agent known for its activity against a wide range of Gram-negative bacteria and Gram-positive bacteria, including MRSA) as a strategy for the control of bacterial adhesion to the mesh. In vitro studies confirmed that low concentrations of CHX, as well as the combination of CHX with allicin, effectively inhibited *S. aureus* adhesion on the surface of the PP mesh soaked in this proposed antimicrobial combination [64].

The efficacy of this new mesh was also tested in vivo, after *S. aureus* inoculation in experimental animals. The in vitro tests for antibacterial activity against *S. aureus* revealed significant inhibition zones for allicin-CHX solution in comparison with CHX alone. However, testing the bacterial colonization on the implant surface showed the poorest behavior for the PP + allicin-CHX group in respect to bacterial load. The proposed modified material exhibited better antimicrobial activity than vancomycin against *S. aureus*, but with the disadvantage of higher fibroblastic destruction. The authors assume that allicin interferes with the inflammatory processes and the macrophage response favoring bacteria survival in the tissue [65].

### 4.3. Surgical Meshes with Antibiotics

**Figure 2:**
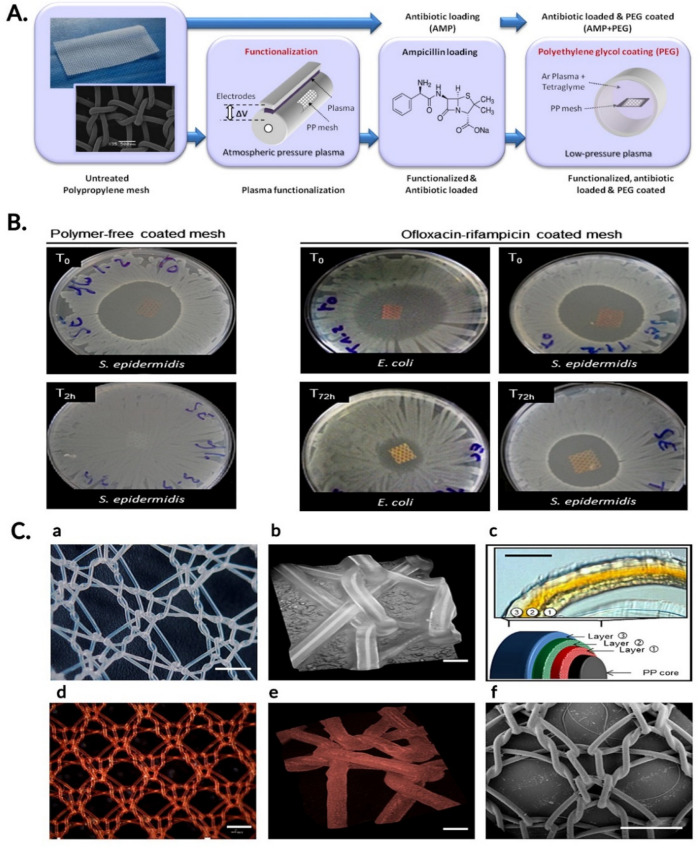
(**A**) Schematic representation of the fabrication process of an ampicillin-loaded and polyethylene (PEG) coated polypropylene surgical mesh. Reprinted with permission from Reference [66]. Copyright 2021 Elsevier. (**B**) The bacterial growth inhibition effect of polymer-free, ofloxacin-rifampicin coated meshes compared to polymer-modified, ofloxacin-rifampicin meshes. The polymer-free mesh presents a quick release of the antibiotic (inhibition not visible after 2 h), while the polymer-modified mesh presents a sustained antibiotic release (inhibition visible after 72 h in both *S. epidermidis* and *E. coli* cultures). Adapted with permission from Reference [85]. Copyright 2021 Elsevier. (**C**) Optical and digital microscope images of the unmodified polypropylene mesh (**a**,**b**) and of the antibiotic (ofloxacin + rifampicin)-coated surgical mesh (**d**,**e**). Cross-sectional image of the three-layer surgical mesh (**c**) and the environmental scanning electron microscope image of the coated surgical mesh (**f**). Reprinted with permission from Reference [85]. Copyright 2021 Elsevier. Created with BioRender.com.

Certain antibiotics, from different classes, were also proposed for prosthetic devices in order to prevent and treat local infections in hernia surgery (Table 3 and Figure 2).

Thus, one of the most known antibiotics, ampicillin, was used to design PP meshes by loading it to the surface of plasma-treated PP monofilaments [66]. The fabrication process of an ampicillin-loaded and polyethylene (PEG)-coated polypropylene surgical mesh is represented schematically in Figure 2A.

The in vitro efficacy and in vivo biocompatibility of an antibiotic polyvinylidenfluoride (PVDF) mesh material using poly(acrylic acid) (PAAc) grafting and subsequent gentamicin binding were tested. The polymeric surface was modified by plasma-induced graft polymerization of acrylic acid and the active sites of the mesh surface were bound with the antibiotic PVDF + PAAc + Gentamicin. After 24 h incubation, the local antimicrobial effects were considered sufficient against the tested bacteria (*S. aureus*, *E. coli*, *S. epidermidis*). In vivo tests (rat models) performed at 7, 21, and 90 days after the mesh implantation showed that neither in vitro cytotoxicity nor in vivo biocompatibility differences were found between the two modified meshes (with or without antibiotic) and the native PVDF mesh [68]. Other in vitro studies of two different meshes impregnated with gentamicin (PE multifilament mesh and a monofilament, partially absorbable PP/poliglecaprone) showed a high bactericidal effect against *S. aureus*, but in vivo studies revealed a low systemic bioavailability [67].

Kilic et al. [69] focused on characterizing cefazolin-impregnated PP meshes. As coating material, a poly(DL-lactide-co-glycolide) solution with cefazolin in dichloromethane was used. Both in vivo and in vitro studies suggested the strong bactericidal activity of cefazolin against different bacterial strains, including MRSA. The results showed a fast release of cefazolin for the first 24 h (followed by a slow release after this time period) and the antimicrobial efficiency of the mesh was proportional to the amount of cefazolin found in each mesh. Furthermore, the in vivo studies showed a relationship between the count of bacteria and the antibiotic concentration (the higher the antibiotic concentration, the lower the count of bacteria isolated from grafts) [69]. The efficacy of absorbable hydrophilic meshes based on polyglycolic acid–trimethylene carbonate (PGA–TMC) impregnated with cefazolin was also evaluated. The results showed a statistically significant decrease in the bacterial colonization (using *S. aureus* inoculum bacteria) for the cefazolin-based meshes compared to the ones without previous antibiotic impregnation [70].

Vancomycin was also used in several studies due to its clinically relevant antibacterial properties and ease of loading into polymers. Thus, Harth et al. [71] proposed a polyester mesh coated with β-cyclodextrin-polyethylene glycol diglycidyl ether loaded with vancomycin for the prevention of *S. aureus* infection. The efficacy of the novel affinity-based drug delivery polymer was demonstrated with the in vivo wound infection model (tests performed at 2 and 4 weeks after implantation). The same group demonstrated in an in vivo pig model study the ability of PE-coated mesh loaded with vancomycin to prevent MRSA infection [73] Moreover, they created a novel coated polymer (microspheres loaded with vancomycin) that was able to decrease the systemic side effects by releasing the antibiotic directly to the desired site of action [72].

Levofloxacin was also used for surgical meshes due to its antibacterial properties. For example, a coated prosthetic material based on PP (surface activated using O_2_ plasma treatment at low pressure) was created using chitosan and levofloxacin. The in vitro studies showed not only significant *S. aureus* and *E. coli* inhibition but also a sustained antimicrobial release for six days [74]. Hall Barrientos et al. proposed polycaprolactone (PCL) electrospun fibers loaded with levofloxacin and their in vitro results highlighted antibacterial capacity against *E. coli* and *S. aureus* [75]. The association of levofloxacin with silver was proposed in order to obtain a new composite material based on poly-L-lactide (PLLA). Using in vitro tests, this combination showed a superior antibacterial efficiency on drug-resistant strains. Moreover, the in vivo studies demonstrated that, when used in combination with low doses of antibiotics, the inhibition growth effect is considered significant for over 8 weeks [76].

Due to the antimicrobial efficiency of ciprofloxacin, a new prosthetic device made of PP functionalized with chitosan and ciprofloxacin coating was proposed. The concentration that provides bactericidal effect against *S. aureus* was established using samples of different concentrations of antibiotics. The kinetics of drugs released is modified according to the chitosan/ciprofloxacin ratios—when this ratio is high, the quantity of antibiotic released is low. The tests in physiological solutions showed that only a part of the antibacterial coating is being dissolved, suggesting that the antibacterial activity could take place both in the biological medium close to the surgical site and also on the device surface [77]. Another prosthetic device with prolonged ciprofloxacin release was obtained by functionalization of the PP mesh with citric acid and hydroxypropyl-γ-cyclodextrin (HPγCD) or maltodextrin (MD). Microbiological assays used *S. aureus*, *S. epidermidis*, and *E. coli* and fibroblasts were used in order to check the tests proliferation and viability. The improved ciprofloxacin absorption/desorption was noted in both modified supports. The modified HPγCD had a lower rate of antibiotic release, confirmed by the microbiological assays, and a decrease in the fibroblast proliferation was noted after 6 days on the modified support [78]. Recently, 3D-printed meshes with ciprofloxacin loading were proposed by Qamar et al. Their personalized 3D-printed meshes (using PP and PVA) showed good mechanical properties. Moreover, in vivo testing (rabbit model) revealed good biocompatibility and faster-wound healing compared to commercial PP mesh [79].

Ofloxacin is another quinolone antibiotic that was successfully employed for the fabrication of antimicrobial meshes. Shokrollahi et al. developed a double-sided surgical mesh, with different properties for the backside and the front side. The backside consisted of PCL and L-DOPA (L-3,4-dihydroxyphenylalanine) which created an adhesive layer so that the mesh cannot be involuntarily displaced. The front layer consisted of either PCL or carboxyethyl-chitosan (CECS) and polyvinyl alcohol (PVA) in which ofloxacin was incorporated. The meshes demonstrated in vitro antibacterial activity against *S. aureus* and especially against *E. coli* [80].

Rifampicin has been widely studied due to its well-known antibacterial properties and common use in the clinic for venous catheters. Thus, a novel antibacterial mesh based on PP, coated with rifampicin-CMC gel was proposed. The in vitro studies showed excellent efficacy against *S. aureus*/*S. epidermidis* for the new prosthetic materials. Compared to the uncoated and CMC gel-coated implants that showed signs of infection and impaired tissue integration, the rifampicin–CMC gel-coated implants exhibited no signs of infection and had optimal tissue integration. Furthermore, the macrophage response was lower in the rifampicin implants compared to the uncoated mesh and to the CMC gel implants. It can be concluded that the composite materials proposed have great efficiency against bacterial adhesion without interfering with tissue repair [81]. Another study evaluated the role of biodegradable poly(lactide-co-glycolide acid)—PLGA—microspheres combined with rifampicin in reducing postoperative PP mesh infections. In vitro data showed *S. aureus* inhibition and the in vivo tests (mouse model) confirmed the reduction in postoperative implant infections by using the new antibacterial mesh with a controlled drug release system [82].

Rifampicin was also proposed in various combinations with antimicrobial properties, with the rifampicin-minocycline combination being well-known and already highly used in clinical practice, especially for preventing venous catheter infections. Thus, a non-crosslinked porcine acellular dermal graft (NCPADG) with a tyrosine polymer coating containing rifampicin and minocycline is now commercially available. The antibiotic role in the coated meshes’ performance was established by comparing the coated versus the uncoated NCPADG after implantation/inoculation in a dorsal rabbit model with either *S. aureus* or *E. coli* [83]. The efficiency of this prosthetic device was confirmed in a multi-institutional retrospective study. The results showed a reduction in occurrences/postoperative complications during the first month of follow-up when the novel rifampicin/minocycline-coated NCPADG was used. There were no negative effects expressed for the patients, for the wound complication rates, or the recurrence rate six months post intervention [84].

Other researchers have used the rifampicin-ofloxacin combination. The available PP mesh contains a dual drug release coating and is made of three layers of antibiotics dispersed in a degradable polymer reservoir made of [poly(ε-caprolactone) (PCL) and poly(DL-lactic acid) (PLA)] (Figure 2C). The polymer ensured the controlled and sustained release of the antibiotics for at least 72 h, thus providing a prolonged antibacterial effect compared to the antibiotic-loaded, polymer-free mesh (Figure 2B). This study also claimed the potential impact of rifampicin and ofloxacin on decreasing the in vitro fibroblast proliferation [85].

Recent studies proposed a new complex combination of four antimicrobial agents—rifampicin, gentamicin, vancomycin, and chlorhexidine. In order to obtain new materials, a polymer solution—hyaluronic acid-poly(N-isopropylacrylamide) (HApN)—was proposed. The hydrogel formed is being used as a coating for meshes when reaching body temperature. The in vitro studies evaluated the performance of this combination for meshes infected with *S. aureus*, *S. epidermidis*, and *E. coli* and the results showed that HApN inhibited both Gram-positive and Gram-negative bacteria growth, with the potential to prevent mesh-related infection [86].

## 5. Perspectives and Conclusions

Surgical meshes are medical devices that can be employed for the consolidation of the abdominal wall in the treatment of hernias. Polypropylene-based surgical meshes are the golden standard option; however, with the rapid development in the biomaterials field, numerous other non-degradable and biodegradable materials are emerging as promising candidates for the development of new surgical meshes. One of the many challenges related to the use of these medical devices is represented by post-operative infection. The presence of infection is correlated with decreased quality of life, increased healthcare costs, and a high socio-economic burden.

In order to prevent surgical site infections, numerous strategies for the development of antimicrobial surgical meshes have been employed. Some commercially available antimicrobial surgical meshes are already used in the clinical setting, while others are only in the experimental stages of development.

Some strategies for the fabrication of antimicrobial surgical meshes include the incorporation of antimicrobial metals, antiseptic substances, or antibiotics into different types of commercially available meshes. The preliminary results obtained with these devices are promising; however, more research needs to be carried out in the field until they can be clinically employed. More specifically, while some surgical meshes have been tested in vivo, most experiments were carried out on animals, with few examples of clinical trials. Extensive clinical trials need to be performed in order to fully assess the biocompatibility and the long-term safety profiles of these surgical meshes in humans, in order to offer more information on the biological behavior of these meshes [7].

As the field of biomaterials continues to progress, it is expected that surgical meshes with improved characteristics, such as high flexibility, tissue biocompatibility, and resistance to shrinkage and adhesion, will be developed in the future.

The methods used for the development of surgical meshes are magnetron sputtering techniques or physical vapor deposition in the case of metallic-coated meshes, impregnation of the commercially available meshes in antiseptic solutions, or the incorporation of antibiotics into polymers or cyclodextrines to obtain antibiotic-based surgical meshes.

In the future, other fabrication techniques such as 3D printing could be employed to develop surgical meshes that are individually tailored to each patient’s needs and that would present improved antibacterial drug release properties [87,88,89].

Another future perspective for the development of antimicrobial surgical meshes consists of the development of antibiotic-based smart meshes [90]. These devices would contain specially designed sensors for infection biomarkers, such as pH, and could trigger antibiotic release only in the presence of bacteria, thus ensuring that treatment is administered only when necessary.

In conclusion, given the important impact of infection on the public health system, numerous strategies have been applied for the development of different types of antimicrobial surgical meshes in this review, these meshes were classified into metallic-coated, antiseptic, and antibiotic-based surgical meshes and their properties were presented and discussed. Silver nanoparticles and other silver compounds were the most commonly employed metallic-based antibacterial agents for the development of metallic-coated surgical meshes, while chlorhexidine was extensively used as an antiseptic. Among antibiotics, vancomycin, beta-lactam antibiotics, and rifampicin were commonly employed for the development of antimicrobial meshes. Despite the need for further tests, especially for clinical trials, these novel medical devices present promising characteristics for the field of surgery. It is expected that 3D printing and the development of smart surgical meshes with incorporated sensors will play an important role in the mitigation of surgical mesh-related infections, thus improving patient quality of life.

## Figures and Tables

**Table 1:** Antimicrobial meshes with metals. NcAg—nanocrystalline silver; AgNP—silver nanoparticles; Ag/SiO_2_—silica/silver layer; PP—polypropylene; Me—metal (Ag, Co, In, W, Zn, Al, Cr, Mn, Ta, Ti); Me-DLC—diamond-like carbon; PEG—polyethyleneglycol; Gel-Hy—gelatin hydrogel; PU—polyurethane; PSIS—porcine-derived small intestinal submucosa; V/TiO_2_—vanadium-doped TiO_2_.

Antimicrobial Agent	Mesh	Test Method	Antibacterial Activity Tested	Ref.
NcAg	PP	In vitro	*S. aureus*	[49]
NcAg	PP	In vivo	MRSA	[50]
AgNP	PP	In vitro In vivo	*E. coli*	[51]
AgNP	PSIS	In vitro In vivo	*S. aureus*, *S. epidermidis*, *E. coli*, *P. aeruginosa*	[48]
Ag/SiO_2_	PP	In vitro	*S. aureus*	[52]
Nano-Ag	PEG/Gel-Hy/PU	In vitro In vivo	*S. aureus*, *E. coli*	[53]
Au, Au-Pd	PP	In vitro In vivo	*S. epidermidis*	[54]
Me/Me-DLC	PP	In vitro	*C. albicans*, *E. coli*, *S. aureus*, *P. aeruginosa*	[55]
Ti	PP	In vitro	*S. aureus*, *P. aeruginosa*, *E. coli*	[56]
Ti	PP	In vitro	*S. epidermidis*, *S. aureus*	[42]
Zn	PP	In vivo	*Enterococcus*, *Staphylococcus*	[57]
V/TiO_2_	PP	In vivo	*S. aureus*, *E. coli*	[58]

**Table 2:** Antimicrobial meshes with antiseptics. CHX—chlorhexidine; Ag-CHX—silver-chlorhexidine complex; QAC—quaternary ammonium compounds; All—allicin; PP—polyproylene; ePTFE—poly-tetrafluoroethylene; CMC—carboxymethylcelulose.

Antimicrobial Agent	Mesh	Test Method	Antibacterial Activity Tested	Ref.
CHX	PP	In vitro In vivo	*S. aureus*	[59]
CHX	CMC	In vitro In vivo	*S. aureus*	[60]
Ag-CHX	ePTFE	In vivo	*S. aureus*	[61]
Ag-CHX	ePTFE	In vitro	MRSA	[44]
Ag-CHX	ePTFE	In vivo	*S. aureus*	[62]
CHX-QAC	PP	In vitro	*S. aureus* *S. epidermidis* *E. coli*	[63]
CHX-All	PP	In vitro	*S. aureus*	[64]
		In vivo		[65]

**Table 3:** Antimicrobial meshes with antibiotics. * vancomycin loaded into cyclodextrine-based polymers; PP—polypropylene; PE—polyester; PCL—polycaprolactone; PGC—poliglecaprone; PVDF—polyvinylidenfluoride; PGA-TMC—polyglycolic acid– trimethylene carbonate; PLLA—poly-L-lactide; PLGA—poly poly(lactide-co-glycolide acid; PADG—porcine acellular dermal graft; PCL—poly(ε-caprolactone); PLA—poly(DL-lactic acid); ePTFE—polytetrafluoroethylene; PSIS—porcine small intestinal submucosa; CHX—chlorhexidine.

Antimicrobial Agent	Meshes	Test Methods	Antibacterial Activity Tested	Ref.
Ampicillin	PP	In vitro	*S. aureus*, *E. coli*	[66]
Gentamicin	PE PP/PGC	In vitro In vivo	*S. aureus*	[67]
Gentamicin	PVDF	In vivo	*S. aureus*, *E. coli*, *S. epidermidis*	[68]
Cefazolin	PE	In vitro In vivo	MRSA	[69]
Cefazolin	PGA–TMC	In vivo	*S. aureus*	[70]
Vancomycin *	PE	In vitro In vivo	*S. aureus*	[71]
Vancomycin *	PE	In vitro	*S. aureus*	[72]
Vancomycin *	PE	In vivo	MRSA	[73]
Levofloxacin Levofloxacin	PP	In vitro	*S. aureus*, *E. coli* *S. aureus*, *E. coli*	[74]
	PCL	In vitro		[75]
Levofloxacin + silver	PLLA	In vitro In vivo	MRSA	[76]
Ciprofloxacin	PP	In vitro	*S. aureus*, *E. coli*	[77]
Ciprofloxacin	PP	In vitro	*S. aureus*, *E*. *coli*, *S. epidermidis*	[78]
Ciprofloxacin	PP, PVC (3D printing)	In vitro In vivo		[79]
Ofloxacin	PCL/L-DOPA and PCL or CECS/PVA	In vitro	*S. aureus*, *E. coli*	[80]
Rifampicin	PP	In vitro In vivo	*S. aureus* *S. epidermidis*	[81]
Rifampicin	PP/PLGA	In vitro In vivo	*S. aureus*	[82]
Rifampicin + Minocycline	PADG	In vitro In vivo	*S. aureus* *E. coli*	[83]
Rifampicin + Minocycline	PADH	In vitro In vivo	MRSA	[84]
Rifampicin + Ofloxacin	PP+ PCL + PLA	In vitro	*E. coli*, *S. aureus*, *S. epidermidis*, *P. aeruginosa*, *K. pneumoniae*, MRSA	[85]
Rifampicin + Gentamicin + Vancomycin + CHX	PP, ePTFE; PGA, PLA, PCL, PSIS, PADG	In vitro	*S. aureus*, *E. coli*, *S. epidermidis*	[86]
